# Perceptions of the causes of schizophrenia and associated factors by the Holy Trinity Theological College students in Ethiopia

**DOI:** 10.1186/s12991-018-0213-3

**Published:** 2018-10-08

**Authors:** Melat Solomon, Telake Azale, Awake Meherte, Getachew Asfaw, Getinet Ayano

**Affiliations:** 1 Institute of Public Health, College of Medicine and Health Sciences, University of Gondar, Gondar, Ethiopia; 2 Research and Training Department, Amanuel Mental Specialized Hospital, Addis Ababa, Ethiopia

## Abstract

**Introduction:**

There is a cultural variability around the perception of what causes the syndrome of schizophrenia. As far as the cause of schizophrenia by the general public concerned, people living in western countries focus mainly on biological and social risk factors such as genetic vulnerability, disease of the brain, infection or stressful social conditions or personal weakness, but the predominant views held by people living in non-western countries focus mainly on supernatural and religious factors. Awareness and beliefs about the causes of mental illnesses influence the preferred treatments. The aim of this study was to determine the perceptions regarding the etiology of schizophrenia and the associated factors by theology students.

**Methods:**

An institution-based cross-sectional study was conducted among Holy Trinity Theological College students from May to June 2016. Self-administered Short Explanatory Model Interview was used to assess the perception of what causes the syndrome of schizophrenia. Data entry was performed by Epi-info version 3.5.3 and the Statistical Program for Social Science (SPSS version 20) was used for data clearance, and analyses.

**Results:**

A total of 409 students were involved in the survey. The mean age of the participants was 33.3 years (standard deviation ± 8.3) and almost all 94.4% of them were males. The majority (81.7%) of the participant recognized schizophrenia as a mental illness. Only 16.9% of the participants attributed supernatural phenomenon as a cause of schizophrenia and most of them 76.5% (313) thought of psychosocial problems as the cause of schizophrenia. About 40.1% of the participant endorsed biological factors as a cause of schizophrenia. About two-thirds (68.2%) of the participant thought schizophrenia as severe but not fatal illness and about 22.2% of them thought both severe and fatal illness. As far as the course concerned majority (88.5%) of the participants thought schizophrenia as a chronic illness and about 11.5% thought acute illness. Regarding the treatment, almost all (99.8%) of reported schizophrenia is treatable. Moreover, concerning the consequences of the illness about 18.8% reported the death as a consequence and about 66.7, 34.7 and 7.8% reported madness, family disintegration and losing a job, respectively. Urban residency and holding other degree were significantly associated with biological factors as a cause of schizophrenia (*p* < 0.05). Whereas getting information from mass media and health professional, marital status (married) and urban residence were significantly associated with psychosocial factors as the cause of schizophrenia. Furthermore, rural residency was significantly associated with the supernatural phenomenon as the cause of schizophrenia.

**Conclusion:**

In the current study, the majority of the participant recognized schizophrenia as a mental illness and a treatable syndrome. A vast majority of the participant thought of psychosocial problems as the cause of schizophrenia about two-thirds of the participant thought schizophrenia as a severe but not fatal illness. As far as the course concerned majority (88.5%) of the participants thought schizophrenia as a chronic illness. Concerning the consequences of the illness, about 18.8% reported the death as a consequence and about 66.7, 34.7, and 7.8% reported madness, family disintegration and losing a job, respectively. Residency, marital status, and source of information were significantly associated with perceived causes of schizophrenia. Linking mental health service with spiritual care to address community mental health care needs and for early detection as well as referral linkage of mentally ill patients is warranted.

## Background

Schizophrenia is defined as a clinical syndrome of the variable, but deeply disruptive, psychopathology that involves cognition, emotion, perception, and other aspects of behavior. The hallmark symptom of schizophrenia is psychosis, such as experiencing auditory hallucinations (voices) and delusions (fixed false beliefs) [1]. Schizophrenia is the most severe among common severe psychiatric disorders. It is among the most disabling and economically catastrophic medical disorders, ranked by the World Health Organization as one of the top ten illnesses contributing to the global burden of disease [2]. There are numerous factors that contribute to the risk of developing schizophrenia. In general, it is a disease caused by a combination of bio-psychosocial influences including genetic, perinatal, and neuroanatomical, neurochemical and other biologic abnormalities [3, 4].

As far as the cause of schizophrenia by the general public concerned, people living in western countries focus mainly on biological and social risk factors such as genetic vulnerability, disease of the brain, infection or stressful social conditions or personal weakness [5–8], but the predominant views held by people living in non-western countries focus mainly on supernatural and religious fact [9–12]. Studies from Australia, Ireland, Germany, Switzerland, UK, and USA have found that social factors were most often seen as the causes of schizophrenia [9–13], whereas genetic factors were much less frequently endorsed [9–11, 13]. Social factors are also seen by the public of Western countries as an important cause of schizophrenia [7, 10, 13, 14]. While genetic factors are more often seen as a cause for schizophrenia than depression, they are still endorsed much less frequently than social factors [9, 13]. In Ethiopia, there is a widespread belief that severe mental illnesses are due to demon possession, bewitchment by an evil spirit and evil eye which existed for many years due to lack of awareness about mental illness [15].

Of greater concern is the stigmatizing belief that mental disorders are caused by personal weakness or a punishment from God. Awareness and beliefs about the causes of mental illnesses influence the preferred treatments [16, 17]. People living in non-western countries mainly rely on indigenous healing practices which are defined as ‘helping beliefs and strategies that originate within a culture or society and that are designed for treating the members of a given cultural group [18]. A research done in Nigeria showed that the majority of people preferred indigenous over the modern health care system which was consistent with their predominant belief about causes of mental illnesses [19]. A study in Ethiopia also showed a preference for indigenous practices for mental disorders and modern medicine is more preferred for somatic conditions [20].

The gap between the need for treatment of mental disorder and its provision is wide all over the world. For example between 76 and 85% of people with severe mental illnesses receive no treatment for their mental health problem [6]. To address the mental health service gaps, integrated management of mental disorders at primary health care level as well as working with traditional healers and spiritual organizations is recommended especially in low- and middle-income countries including Ethiopia. However, there is a gap with respect to collaboration with the faith and traditional healers to create awareness and promote modern mental health care [21].

While there has been considerable research on public beliefs, there has been little research concerning the belief of religious leaders including theology students. Therefore, in view of the above issues, the aim of this study was: (1) to explore the perceived causes of schizophrenia by theology students; (2) to identify the factors associated with perceived causes of schizophrenia.

## Methods

### Study setting and design

Institutional-based cross-sectional study was conducted at Holy Trinity Theological College, May 2016, Addis Ababa, Ethiopia. Holy Trinity Theological College (HTTC) is a theological school of higher education located in Addis Ababa, Ethiopia. The college provides religious and secular instruction to both clergies and lay members of the Ethiopian Orthodox Church. In addition, the institution serves as a center of theological and ecclesiastical study for all Oriental Orthodox Churches. The college gives both undergraduate and postgraduate training and the trained professionals are expected to work as spiritual teachers and also expected to involve in different managerial positions of the Ethiopian Orthodox church. The graduates of the Holy Trinity Theological College are the most expected peoples in Ethiopia because they are assumed to be a bridge between the people and God. The Ethiopian Church has a membership of between 40 and 46 million, the majority of whom live in Ethiopia, and is, thus, the largest of all Oriental Orthodox churches.

### Study population

The study population consisted of all students who were attending Holy Trinity Theological College and included in the sample. Those who are unable to communicate due to different reasons were excluded from the study.

### Sampling procedures

The sample size was calculated using Epi-info software version 3.5.2 by considering the following assumptions: 95% Confidence Interval, 80% power, 50% proportion of etiology of schizophrenia, odds ratio to be detected 2, Non-response rate 10%. The final sample size was 423. Simple random sampling technique was used to select the study participants.

### Data collection

The data were collected by semi-structured, self-administered Short Explanatory Model Interview (SEMI) version 3.2 It is developed by Lloyd et al. [22] and modified by different psychiatrists to Ethiopian version to measure perception with modification to make it suitable for non-patients that were respond based on case vignettes. The questionnaires were including socio-demographic characteristics, clinical factors, and source of information. Questionnaires were asked after reading the vignette case. As recommended by WHO a case vignette study has also been used in a study in Ethiopia to evaluate mental health training and its implication for integrated mental health services [23], a study Butajira to assess attitude about mental disorders [20], on Ethiopian medical students [24], and as part of the national mental health plan in the United Republic of Tanzania [25], and to assess perception and attitude of the community about mental disorders in Ethiopia [26].

### Data processing and analyses

Data were analyzed using SPSS version 20. Frequency and percentage were used to describe the data. Bivariate analysis was done to see the association of each independent variable with the outcome variable. Those variables having a *p* < 0.2 were entered into the multivariate logistic regression model to identify the effect of each independent variable with the outcome variables. A *p* < 0.05 was considered statistically significant, and the adjusted odds ratio with 95% CI was calculated to determine the association.

### Ethical consideration

Ethical clearance was obtained from the University of Gondar Ethical Review Board (ERB) and Amanueal Mental Specialized Hospital Ethical Review Board. Informed consent was obtained from each participant during data collection. All participants were informed about the aim and purpose of the study. The right was given to the study participant to refuse and stop or withdraw from participation at any time during data collection without loss of any entitlement. For the purpose of anonymity, participants name was not used at the time of data collection and all other personnel information was kept entirely anonymous and confidential.

## Results

### Socio-economic and demographic characteristics

A total of 409 participants were included in the study which makes the response rate 96.7%. The mean age of the respondents was 33.3 (SD ± 8.3) and almost all 386 (94.4%) were males. About 184 (45.0%) of the participants were Amhara by ethnicity. More than half 223 (54.5%) were single and 186 (45.5%) married. About 215 (52.6%) of participants were from urban, the remaining 194 (47.4) came from rural areas. Regarding previous college training of the participants, about 167 (40.8%) respondents have held a diploma and other degrees; of these 83 (20.1%) had training related to business and construction profession, about 51 (12.5%) had training related to health, and 35 (8.6%) were teachers. Moreover, about 159 (38.9%) of the participants had a diploma-level training, 227 (55.5%) had a degree-level training, and 23 (5.6%) had master’s degree (see Table 1).

**Table 1:** Frequency of socio-demographic characteristics of Holy Trinity Theological College students at Addis Ababa Ethiopia 2016 (*n* = 409)

Variable	Frequency	Percentage
Age		
Mean (SD)	33.3 (8.3)	
Sex		
Male	386	94.4
Female	23	5.5
Ethnicity		
Amhara	184	45.0
Tigre	92	22.5
Oromo	49	12.0
Gurage	54	13.2
S.N.N.P	30	7.3
Marital status		
Single	223	54.5
Marred	186	46.1
Residency		
Urban	215	52.6
Rural	194	47.4
Educational level		
Diploma	159	38.9
Degree	227	55.5
Masters	23	5.5
Background profession		
Yes	167	40.8
No	242	59.1
Type of profession		
Business and construction	83	20.1
Health profession	51	12.5
Teaching	35	8.6

### Clinical factors and source of information of respondents

Among the total participants, 111 (22.1%) of respondents were reported to have a family history of mental illness and about 13 (3.2%) had a previous mental illness. Regarding the source about mental illness, all respondents reported as having mental illness-related information; from this, 322 (78.7) heard the information from social media (see Table 2).

**Table 2:** Frequencies of family history of mental illness, previous history of mental illness and sources of information among Holy Trinity Theological College students Addis Ababa Ethiopia 2016

Variable	Frequency	Percentage
Family history of mental illness		
No	298	27.1
Yes	111	72.9
Previous mental illness		
No	396	96.8
Yes	13	3.2
Sources of information		
Social media	322	78.7
Mass media	241	58.9
Health professional	196	47.9

### Perceived cause, course, outcomes and treatment of schizophrenia by participants

The majority (81.7%) of the participant recognized schizophrenia as a mental illness. Only 16.9% of the participants attributed supernatural phenomenon as a cause of schizophrenia and most of them 76.5% (313) thought of psychosocial problems as the cause of schizophrenia. About 40.1% of the participant endorsed biological factors as a cause of schizophrenia. About two-thirds (68.2%) of the participant thought schizophrenia as severe but not fatal illness, and about 22.2% of them thought both severe and fatal illness.

As far as the course concerned, majority (88.5%) of the participants thought schizophrenia as a chronic illness and about 11.5% thought acute illness. Regarding the treatment, almost all (99.8%) of reported schizophrenia is treatable. Moreover, concerning the consequences of the illness, about 18.8% reported the death as a consequence and about 66.7, 34.7 and 7.8% reported madness, family disintegration and losing a job, respectively (see Table 3).

**Table 3:** Frequency of perceived cause of schizophrenia by Holy Trinity theological college students Addis Ababa 2016

Variable	Schizophrenia	
	Frequency	Percentage
Name		
Non specific psychi/psycho dx	318	77.8
Unable to say	55	13.4
Supernatural/spiritual	18	4.4
Specific psychi/psycho dx	16	3.9
Non specific physiology dx	1	0.2
Specific physiological dx	1	0.2
Cause		
Psychosocial	313	76.3
Supernatural/spiritual	90	16.9
Biological	61	14.9
Severity		
Severe but not fatal	278	68.2
Severe and fatal	91	22.2
Moderate	32	7.8
Mild	8	2.0
Course of illness		
Chronic	362	88.5
Acute	46	11.2
Life long	1	0.02
Treatment		
Yes	409	100.
No	0	
Treatment option		
Psychosocial	280	68.5
Spiritual/traditional	194	47.4
Biological	164	40.1
Treatment outcome		
Treatable	408	99.8
Not treatable	1	0.2
Consequence		
Madness	278	66.7
Family disintegration	142	34.7
Death	77	18.8
Loosing job	32	7.8

*DX* diagnosis, *Psycho* psychological, *Psychi* psychiatric

### Factors associated with the perceived cause of schizophrenia among Holy Trinity Theological College students

During multivariable analysis, variables residency, having another profession, marital status and source of information were significantly associated with perceived causes of schizophrenia.

In our multivariate logistic regression, individuals who were from the urban area were strongly associated [AOR = 3.34 (1.63, 6.80)] with perceived biological causation. Those having other background profession were strongly and positively associated with the perceived biological cause of schizophrenia with [AOR = 5.17 (2.67, 10.23)]. Residency (urban) was also positively associated with the perceived psychosocial cause of schizophrenia with [AOR = 1.72 (1.03, 2.86)]. Respondents those who are married were associated with the psychosocial cause of schizophrenia with (AOR = 0.46 (0.25, 0.866). Getting information about mental illness from mass media [AOR = 1.999 (1.22, 3.26)] and from a health professional [AOR = 1.888 (1.158, 3.07)] were associated with the perceived psychosocial cause of schizophrenia. Perceived supernatural/spiritual cause of schizophrenia was strongly associated with those who came from rural residency with [AOR = 3.107 (1.8, 5.314)] (See Table 4).

**Table 4:** Bivariate and multivariate analysis of factors associated with perceived cause of schizophrenia by Holy Trinity Theological College students Addis Ababa Ethiopia 2016

Variables	Categories	Biological		COR (95% CI)	AOR (95% CI)
		No	Yes		
Age				1.07 (0.98, 1.049)	1.99 (0.96, 1.03)
Marital status	Single	191	32	1.00	
	Marred	157	29	1.13 (0.63, 1.9)	
Residency	Urban	165	50	5.04 (2.53, 10.1)	3.33 (1.63, 6.8)***
	Rural	183	11	1.00	1.00
Background profession	Yes	119	48	7.1 (3.7, 13.6)	5.17 (2.67, 10.23)***
	No	229	13	1.00	1.00
Family Hx of mental illness	Yes	95	16	0.9 (0.5, 1.75)	
	No	253	45	1.00	
Previous Hx of mental illness	Yes	11	2	1.03 (0.22, 4.80)	
	No	337	59	1.00	
Sources of information	Social media				
	Yes	268	54	0.77 (0.44, 1.34)	0.27 (0.73, 4.25)
	No	80	7	1.00	1.00
	Mass media				
	Yes	202	39	1.28 (0.72, 2.25)	
	No	146	22	1.00	
	Health professional				
	Yes	170	26	2.33 (1.08, 5.26)	
	No	178	35		

Variables	Categories	Psychosocial		COR (95% CI)	AOR (95% CI)
		No	Yes		
Age				0.9 (0.9, 0.99)	0.98 (0.9, 1.03)
Marital status	Single	40	183	1.00	1.00
	Marred	56	130	0.50 (0.3, 0.8)	0.46 (0.25, 0.86)*
Residency	Urban	38	117	1.98 (1.24.3.16)	1.72 (1.03, 2.86)*
	Rural	58	136	1.00	1.00
Background profession	Yes	32	135	1.51 (0.93, 2.45)	1.43 (0.83, 2.46)
	No	64	178	1.00	1.00
Family Hx of mental illness	Yes	27	84	0.93 (0.56, 1.56)	
	No	69	229	1.00	
Previous Hx of mental illness	Yes	4	4	0.68 (0.20, 2.26)	
	No	92	304	1.00	
Sources of information	Mass media				
	Yes	46	195	1.79 (1.33, 2.85)	1.99 (1.22, 3.26)**
	No	50	118	1.00	1.00
	Social media				
	Yes	73	249	1.26 (0.72, 2.11)	
	No	23	64	1.00	
	Health profession				
	Yes	35	161	1.89 (1.15, 2.95)	1.88 (1.15, 3.07)*
	No	61	152	1.00	

Variables	Categories	Supernatural/spiritual		COR (95% CI)	AOR (95% CI)
		No	Yes		
Age				1.07 (0.9, 1.04)	1.07 (0.98, 1.05)
Marital status	Single	179	44	1.33 (0.83, 2.13)	0.78 (0.41, 1.48)
	Marred	140	46	1.00	1.00
Residency	Urban	189	26	1.00	1.00
	Rural	130	64	3.57 (2.15, 5.9)	3.17 (1.8, 5.34)***
Background profession	Yes	142	25	1.00	1.00
	No	177	65	2.08 (1.25, 3.47)	1.58 (0.9, 2.77)
Family Hx of mental illness	Yes	86	25	1.00	
	No	233	65	0.96 (0.56, 1.62)	
Previous Hx of mental illness	Yes	11	2	1.00	
	No	308	88	1.57 (0.34, 7.22)	
Sources of information	Mass media				
	Yes	194	47	1.00	1.00
	No	125	43	1.420 (0.88, 2.274)	1.49 (0.91, 2.45)
	Social media				
	Yes	253	69	1.00	
	No	60	21	1.16 (0.66, 2.040)	
	Health professional				
	Yes	160	36	1.00	1.00
	No	159	54	1.59 (0.93, 2.42)	0.78 (0.41, 1.48)

* *p* < 0.05, ** *p* < 0.01, *** *p* < 0.001

## Discussion

To the best of our knowledge, this is the first study which examined the perceived cause of schizophrenia, and associated factors among Holy Trinity Theological College students. The study revealed that only 16.9% of the participants attributed supernatural phenomenon as a cause of schizophrenia and most of them 313 (76.5%) reported psychosocial problems as the cause of schizophrenia. The findings of this study were different than the results from the previous studies in Nigeria which showed the perceived cause of schizophrenia to be supernatural in 93.8% [27] and study done in Ethiopia where most frequently mentioned cause of mental illness spirit possession 51.6% [28]. This could be the difference between study participants. Since they are theological college students and they had the basic academic background. The other possible cause could be due to more than half respondents are from urban dwelling; this might help them to better access to getting information. Another reason may be that the psychology course is included in their curriculum.

In the current study, majority (81.7%) of the participant recognized schizophrenia as a mental illness. This could be due to almost all respondents have mental health information. The other possible explanation may be due to its symptoms of schizophrenia are observable in nature or are overt.

Schizophrenia was regarded as severe and but not fatal by the majority of the respondents 278 (68.25). It was also regarded as chronic mental disorders by the majority of the respondents 362 (88.5%). This is in agreement with a study conducted in Ethiopia [29].

Concerning perceived treatments, 280 (68.5%) of respondents preferred psychosocial management for schizophrenia. Almost all participants agreed with the treatment outcome as treatable. This may be due to psychology course is included in their curriculum. These results are in harmony with other studies conducted in Ethiopia [29].

Residency is found to be a significant factor that associates with the perceived psychosocial cause of schizophrenia. Respondents with urban residency were found associated with psychosocial and biological perceived cause for schizophrenia. Respondents with rural residency were found associated with supernatural/spiritual perceived cause for schizophrenia disorder. This result is in agreement with the study done in Nigeria regarding the cause of mental illness where urban dwelling was found to be correlated with beliefs in biological and psychosocial causation but rural dwelling was found correlated with beliefs in supernatural causation [29].

The present study found an association between perception about schizophrenia and the source of information. Getting information from mass media and from health professionals was positively associated with the perceived psychosocial cause of schizophrenia. This result is in agreement with results from similar studies conducted in Ethiopia, Hawassa they reported information from mass media and health professionals has good perception towards mental illness [26].

In this study, having another background profession (diploma and degree) showed significant association with perceived biological and psychosocial cause of schizophrenia. On the other hand, not having other background profession (previous training college training) showed significant association with the perceived supernatural cause of schizophrenia. This might be due to better access to information through another profession. This result cannot be compared with other studies because no study was identified which tried to assess the perception of specific mental disorders as well as consider another background profession as a variable.

The result of this study revealed that marital status was significantly associated with perceived psychosocial causes of schizophrenia. Participants who are married were significantly associated with perceiving the psychosocial cause of schizophrenia. This result cannot be compared with other studies because no study was identified which tried to consider marital status as a variable for each specific mental disorder.

In this study, perceived cause of respondents was not found significantly associated with age, family history of mental illness and previous history of mental illness.

## Conclusion

In the current study, the majority of the participants recognized schizophrenia as a mental illness and a treatable syndrome. Most of the participants thought of psychosocial problems as the cause of schizophrenia. About two-thirds of the participant thought schizophrenia as severe but not a fatal illness. Most of the participants (88.5%) thought schizophrenia as a chronic illness and about 18.8% reported schizophrenia results in the death. Moreover, 66.7, 34.7, and 7.8% reported madness, family disintegration and losing a job, respectively, as an outcome. In this study, residency, marital status, and source of information were significantly associated with perceived causes of schizophrenia. Linking mental health service with spiritual care to address community mental health care needs and for early detection as well as referral linkage of mentally ill patients is warranted.
